# Optimization of school physical education schedules to enhance long-term public health outcomes

**DOI:** 10.3389/fpubh.2025.1548056

**Published:** 2025-02-19

**Authors:** Sun Tao, Zhu Sheng-ping, Wang Meng-yuan

**Affiliations:** ^1^School of Physical Education, Hunan University of Arts and Science, Changde, China; ^2^Teaching Affairs Office, Xikou Middle School, Zhangjiajie, China

**Keywords:** long-term public health outcomes, digital health, optimization, physical education schedules, fitness improvement

## Abstract

**Introduction:**

Optimizing school physical education (PE) schedules is crucial for enhancing public health outcomes, particularly among school-aged children.

**Methods:**

Therefore, in this study, a weighted fitness function is developed to evaluate health fitness scores. This function integrates multiple health metrics such as BMI reduction, fitness improvement, calories burned, and heart rate reduction. Six optimization algorithms such as Genetic Algorithm (GA), Particle Swarm Optimization (PSO), Ant Colony Optimization (ACO), Simulated Annealing (SA), Differential Evolution (DE), and Artificial Bee Colony (ABC) optimization algorithms are utilized to optimize PE schedules based on the designed weighted fitness function. Using a dataset of 1,360 student entries, the study incorporates health metrics such as BMI reduction, fitness score improvement, caloric expenditure, and heart rate reduction into a weighted fitness function for optimization.

**Results:**

The results show that ACO achieved the highest allocation of PE time (9.91 h/week), the most significant caloric expenditure (370 kcal/session), and the greatest reduction in heart rate (8.5 bpm). GA excelled in the reduction of BMI, achieving a decrease of 10.63 units.

**Discussion:**

These analyses reveal the transformative potential of optimized PE schedules in reducing the burden of lifestyle-related diseases, promoting equitable health outcomes, and supporting cognitive and mental well-being. Finally, recommendations are provided for policymakers and stakeholders to implement data-driven PE programs that maximize long-term public health benefits.

## 1 Introduction

### 1.1 Background and rationale

Physical education (PE) plays a crucial role in promoting physical, mental, and social wellbeing, particularly among school-age children. However, with the increasing prevalence of sedentary lifestyles and associated health risks, the optimization of PE programs has become an urgent need to improve public health outcomes. Schools, as foundational educational institutions, are uniquely positioned to provide structured opportunities for physical activity and health education (1).

The effectiveness of PE programs depends significantly on their design and implementation. Research emphasizes that PE programs need to be data-driven, equitable, and adaptive to meet the diverse needs of students (2, 3). Traditional approaches to scheduling PE often rely on arbitrary time allocations and outdated teaching methodologies, which do not maximize the health benefits of these sessions (4, 5). Studies suggest that incorporating optimization techniques into PE scheduling can improve resource allocation and time to align with global public health goals, such as the World Health Organization recommendation of at least 60 minutes of moderate to vigorous physical activity daily (6).

### 1.2 Related work

Recent advances in technology and computational methods have introduced optimization algorithms into the field of education, enabling more effective scheduling and resource utilization. Algorithms such as Genetic Algorithm (GA), Particle Swarm Optimization (PSO), and Ant Colony Optimization (ACO) have been successfully applied to improve the efficiency of PE programs and ensure alignment with health outcomes (7, 8). These approaches integrate factors like activity intensity, session frequency, and individual health metrics to deliver personalized and impactful PE schedules (9).

Moreover, the incorporation of technology in PE programs, including wearable devices and big data analytics, has further enhanced the ability to monitor, assess, and optimize physical activity levels (10, 11). These innovations not only improve the accuracy of health assessments but also facilitate the implementation of targeted interventions, particularly for students with varying fitness levels or health conditions (12). Various studies have emphasized the importance of structured and optimized PE schedules in promoting critical health outcomes such as BMI reduction, fitness improvement, caloric expenditure, and cardiovascular health (13, 14).

### 1.3 PE schedules for public health impact

Despite the growing recognition of PE's role in public health, traditional scheduling approaches fail to leverage the advancements in optimization techniques and data analytics. These conventional methods often rely on arbitrary time allocations and generic strategies, resulting in suboptimal use of resources and limited impact on student health (15, 16). Furthermore, the diversity in student demographics and health needs necessitates adaptive, personalized schedules to maximize the health benefits of PE programs (15, 16). Recent advances in computational methods, such as Genetic Algorithms (GA), Particle Swarm Optimization (PSO), and Ant Colony Optimization (ACO), provide powerful tools to address these challenges by enabling the design of schedules that are efficient, equitable, and outcome-focused (4, 6).

### 1.4 Research motivation

Motivated by the need for more effective PE programs and the potential of optimization algorithms to improve scheduling, this study aims to develop and evaluate methodologies that can maximize health outcomes. By integrating health metrics with scheduling features, this study seeks to provide insights for educators, policymakers, and public health professionals. The primary objective of this study is to design and evaluate optimized PE schedules using optimization techniques to enhance long-term public health outcomes.

Additionally, optimized PE schedules have the potential to improve both mental health and academic performance among students. Regular physical activity has been shown to reduce stress, anxiety, and symptoms of depression. These benefits contribute to enhancing overall mental wellbeing. Furthermore, improved physical fitness is closely linked to better cognitive function, memory, and attention. These cognitive benefits are critical for achieving academic success. By promoting structured and effective PE programs, this study aims to create an environment that supports physical health while fostering mental and academic development.

### 1.5 Contributions

The key contributions of this paper are as follows:

1. A weighted fitness function is developed to evaluate health fitness scores. This function integrates multiple health metrics such as BMI reduction, fitness improvement, calories burned, and heart rate reduction.
2. The Six selected optimization algorithms such as Genetic Algorithm (GA), Particle Swarm Optimization (PSO), Ant Colony Optimization (ACO), Simulated Annealing (SA), Differential Evolution (DE) and Artificial Bee Colony (ABC) optimization algorithms are utilized to optimize PE schedules based on the designed weighted fitness function.
3. Finally, this study highlights various public health implications of optimized PE schedules and also provides recommendations to policy makers and stakeholders to implement PE programs in schools to address health disparities and improve resource utilization.

## 2 Background on PE programs and health metrics

In schools, PE programs are designed to improve students' physical, mental, and social wellbeing. These programs incorporate various structured activities that promote physical fitness, develop motor skills, and encourage teamwork and social interaction (17, 18).

### 2.1 Typical PE program contents

PE programs typically include aerobic exercises, strength training, team sports, flexibility training, and recreational activities. Each component serves a specific purpose, from improving cardiovascular endurance to fostering team-building skills (19). Table 1 outlines these components along with their descriptions and examples.

**Table 1:** Typical PE program contents.

**Component**	**Description**	**Examples**
Aerobic exercises	Activities to improve cardiovascular endurance.	Running, cycling, jumping
Strength training	Exercises focused on building muscle strength and endurance.	Push-ups, squats, resistance band training
Team sports	Group activities to develop teamwork and coordination.	Basketball, soccer, volleyball
Flexibility training	Exercises to enhance range of motion and prevent injuries.	Yoga, stretching routines
Recreational activities	Fun and engaging activities to encourage participation and enjoyment.	Obstacle courses, group challenges

### 2.2 Alignment with institutional guidelines

PE programs must align with guidelines from institutions like the World Health Organization (WHO) and local educational authorities to ensure they are effective and feasible (20). These guidelines emphasize the importance of structured physical activity tailored to students' age groups.

For younger children (ages 6–10), activities should focus on fun and motor skill development (21). Pre-adolescents (ages 11–13) benefit from structured aerobic and strength training. The adolescents (ages 14–18) require fitness improvement and sports-specific skills. Table 2 summarizes the alignment of PE activities with age groups and institutional recommendations.

**Table 2:** Alignment of PE activities with age groups and guidelines.

**Age group**	**Focus areas**	**Activities**
6–10 years	Fun, motor skill development	Games, running, simple obstacle courses
11–13 years	Structured aerobic and strength training	Jogging, resistance training
14–18 years	Fitness improvement, sports-specific skills	Team sports, endurance exercises

### 2.3 Suitability of health metrics

To evaluate the effectiveness of PE programs, several health metrics are used. These metrics assess physical, physiological, and fitness outcomes, providing a comprehensive understanding of student health (22). Table 3 lists these metrics along with their definitions, significance, and appropriate use cases (see Section 3.4 for mathematical definitions).

**Table 3:** Health metrics for evaluating PE programs.

**Health metric**	**Definition**	**Significance**	**Use case**
BMI	A measure of weight status based on height and weight.	Tracks obesity and weight management.	Useful for all age groups.
Fitness score	A composite score of physical fitness, including endurance and strength.	Tracks progress in physical capabilities.	Suitable for structured fitness goals
Calories burned	Energy expenditure during physical activities.	Indicates activity intensity and effectiveness.	Relevant for aerobic exercises.
Heart rate reduction	Change in resting heart rate due to regular physical activity.	Reflects cardiovascular health improvement.	Best for adolescents.

### 2.4 Significance in assessing PE programs

These health metrics have been widely used to evaluate the effectiveness of PE programs (23, 24). Metrics such as BMI is essential for monitoring and managing childhood obesity. It offers valuable insights into students' weight status and overall health (25). Fitness scores assess physical capabilities like endurance and strength. These scores help identify areas for improvement and guide targeted interventions to enhance fitness levels (26). Additionally, tracking calories burned during activities measures the intensity and effectiveness of exercises. This ensures that students achieve energy balance and meet their physical activity goals (27). Reductions in resting heart rate serve as a key indicator of cardiovascular health. They highlight the long-term benefits of consistent physical activity (28). By aligning these metrics with institutional guidelines, schools can design PE programs that address students' diverse needs. Such alignment ensures immediate physical and mental health improvements. It also promotes lifelong wellness and the development of healthy habits (29).

## 3 Methodology

**Figure 1:**
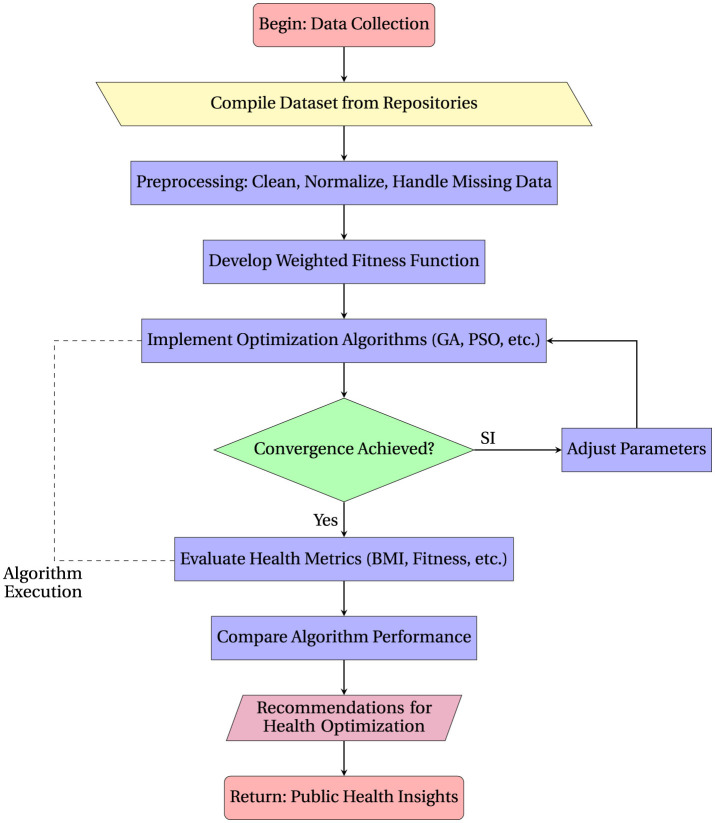
Flowchart of the optimization process to enhance public health outcomes.

This section outlines the approach adopted in the study, detailing the methods used for data collection, the implementation of optimization algorithms, and the evaluation of health metrics. Figure 1 shows the diagrammatic flow of the optimization process for enhancing public health outcomes.

### 3.1 Data collection

The dataset for this study was compiled from publicly available educational and health repositories, as well as anonymized student records collected from multiple schools. To ensure the dataset was suitable for applying optimization algorithms, we focused on collecting comprehensive and diverse data that captures both individual health metrics and PE program parameters. The final dataset consists of 1,360 entries, representing a diverse sample of students from various demographic and geographic backgrounds.

#### 3.1.1 Open data sources

The data used in this study were sourced from the following repositories, chosen for their detailed and structured records relevant to health and physical activity:

1. **Gym-exercise-data-analysis repository:** provides granular data on physical activity patterns, including types of activities, durations, and associated health metrics (30).
2. **CDC data: nutrition, physical activity, and obesity:** offers extensive data on nutrition, activity levels, and obesity-related metrics across various demographics in the United States (31).
3. **New Jersey State health assessment data:** focuses on physical activity behaviors and health outcomes of school-age children, including BMI, activity levels, and cardiovascular health (32).

#### 3.1.2 Dataset features

To build an adequate dataset for applying optimization algorithms, we included features that represent both individual health parameters and the operational details of PE programs. These features are essential for optimizing schedules to improve specific health outcomes. They are grouped into two categories:

**A. Health-Related Features:** These metrics capture the baseline health status and outcomes of individual students:

- **Age and Gender:** Demographic data to analyze health outcomes across diverse groups.
- **BMI:** Baseline Body Mass Index to assess weight status and its changes.
- **Fitness Score:** Initial scores from standardized fitness tests to evaluate endurance and strength.
- **Resting Heart Rate (RHR):** A measure of cardiovascular health.
- **Caloric Expenditure:** Calories burned during PE sessions, calculated using activity intensity and duration.

**B. PE Schedule-Related Features:** These variables describe the structure and intensity of PE programs:

- **Weekly PE Time:** Hours allocated for PE activities in schedules.
- **Activity Distribution:** Time spent on aerobic exercises, strength training, team sports, etc.
- **Activity Intensity Levels:** Categorized as low, moderate, or vigorous based on MET values.
- **Session Frequency:** Number of PE sessions per week.
- **Instructor-to-Student Ratio:** A measure of instructional quality.
- **Facility Utilization Rate:** Percentage of PE facilities used during sessions.

#### 3.1.3 Rationale for dataset features

The features selected for this dataset are specifically designed to support the optimization process. Health-related metrics, such as BMI and fitness score, serve as key indicators of program outcomes. PE schedule-related features provide the necessary inputs for optimization algorithms to design effective schedules. This combination ensures that the dataset aligns with the goals of improving health outcomes and maximizing resource utilization.

### 3.2 Data refinements

To ensure the dataset's reliability and suitability for algorithmic analysis, a series of data preprocessing steps were undertaken. Each approach was carefully selected based on best practices and supported by relevant literature.

#### 3.2.1 Handling missing data

Missing data can compromise the integrity of analysis and introduce bias. To address this, continuous variables, such as age and BMI, were imputed with mean values, ensuring that the central tendency of the data was preserved (33). Categorical variables, like activity levels or gender, were completed using mode imputation, which maintains the most frequent category's representation. These methods are widely used due to their simplicity and ability to minimize distortions in the dataset (34).

#### 3.2.2 Outlier detection

Outliers, which can skew analysis and lead to erroneous conclusions, were identified and managed using the interquartile range (IQR) method. Values outside 1.5 times the IQR from the first and third quartiles were flagged as outliers and subsequently removed (35). This approach is particularly effective in datasets with a non-Gaussian distribution and ensures that the remaining data points are representative of typical observations (36).

#### 3.2.3 Normalization

Continuous variables, such as BMI and fitness scores, were normalized using min-max scaling to bring all values into a common range. Normalization ensures that variables with larger ranges do not dominate the learning process in algorithms sensitive to magnitude differences (37). The min-max scaling can be computed using Equation 1:


(1) $$\begin{array}{l} x_{\text{normalized}}=\frac{x-x_{\text{min}}}{x_{\text{max}}-x_{\text{min}}} \end{array}$$

This technique is particularly advantageous for algorithms such as gradient descent, where scale invariance is crucial (38).

#### 3.2.4 Encoding categorical features

To prepare categorical variables like gender or activity types for algorithmic processing, one-hot encoding was applied. This technique transforms categorical data into binary vectors, ensuring compatibility with machine learning models that require numerical inputs (39). For example, a gender variable with categories “Male” and “Female” would be represented as [1, 0] and [0, 1], respectively. One-hot encoding prevents algorithms from assuming ordinal relationships between categories (40).

### 3.3 Final dataset

The final refined dataset includes 1,360 entries, with a balanced representation across age groups and genders, ensuring diversity and applicability of results. Table 4 provides a summary of the features included in the dataset.

**Table 4:** Summary of Features in the Dataset.

**Feature**	**Description**	**Type**
Age	Age of the student (6–18 years)	Continuous
Gender	Gender of the student (male/female)	Categorical
BMI	Baseline Body Mass Index	Continuous
Fitness score	Initial fitness score (scale 0–100)	Continuous
PE time	Weekly PE hours allocated	Continuous
Activity distribution	Time proportion for different PE activities	Continuous
Activity intensity level	Categorized as low, moderate, or vigorous	Categorical
Resting heart rate (RHR)	Baseline average heart rate (bpm)	Continuous
Caloric expenditure	Estimated calorie burn during PE sessions	Continuous
Session frequency	Number of PE sessions per week	Continuous
Instructor-to-student ratio	Students supervised by one instructor	Continuous
Facility utilization rate	Percentage of available PE facilities utilized	Continuous

### 3.4 Health metrics

The effectiveness of each optimization algorithm was evaluated based on the following key health metrics. These metrics were chosen to assess the impact of optimized PE schedules on public health outcomes, including physical fitness, weight management, and cardiovascular health. Namely, these metrics are BMI reduction (Equation 2), fitness improvement (Equation 3), calories burned (Equation 4), basal metabolic rate (BMR) (Equation 5) and heart rate reduction (Equation 6). The weighted fitness function is represented by Equation 7.

#### 3.4.1 BMI reduction

Body Mass Index (BMI) (41) is a widely used measure to classify weight status and assess weight-related health risks. BMI reduction was calculated as:


(2) $$\begin{array}{l} \Delta \text{BMI}={\text{BMI}}_{\text{initial}}-{\text{BMI}}_{\text{final}}, \end{array}$$

where BMI_initial_ and BMI_final_ are the BMI values before and after implementing the optimized PE schedule. A higher ΔBMI indicates a more significant improvement in weight management.

#### 3.4.2 Fitness improvement

Fitness improvement reflects enhancements in cardiovascular and muscular endurance. It was quantified using a standardized fitness score derived from physical fitness tests, including endurance runs and strength exercises:


(3) $$\begin{array}{l} \Delta F=F_{\text{final}}-F_{\text{initial}}, \end{array}$$

where *F*_final_ and *F*_initial_ are the fitness scores after and before the implementation of the optimized schedules. A higher Δ*F* indicates better improvements in physical fitness.

#### 3.4.3 Calories burned

Calories burned during PE sessions is a measure of energy expenditure, which contributes to weight loss and overall fitness. To enhance accuracy and account for individual metabolic differences, the caloric expenditure formula was updated to include a factor for Basal Metabolic Rate (BMR), as well as individualized metabolic variations based on age, gender, and fitness level. The enhanced formula is as follows (42):


(4) $$\begin{array}{l} \text{Calories Burned}=\text{MET}\cdot W\cdot t\cdot \left(1+\frac{\text{BMR}}{\text{1000}}\right), \end{array}$$

In this formula, MET represents the metabolic equivalent of task for a given activity, such as running or strength training. *W* denotes the student's weight in kilograms, while *t* indicates the duration activity in hours. BMR is the Basal Metabolic Rate, which accounts for individual metabolic variations and is calculated as:


(5) $$\begin{array}{l} \text{BMR}=10\cdot W+6.25\cdot H-5\cdot A+S, \end{array}$$

where *H* is the height of the individual in centimeters, *A* is the age in years, and *S* is a gender-specific constant (+5 for males and −161 for females).

BMR adjusts the caloric expenditure formula to better reflect individual differences in metabolism. It improves the precision of energy expenditure calculations and makes them more suitable for diverse student populations.

#### 3.4.4 Heart rate reduction

Resting heart rate (RHR) is a strong indicator of cardiovascular health. A reduction in RHR signifies improved heart function and endurance. The change in heart rate is calculated as:


(6) $$\begin{array}{l} \Delta HR=HR_{\text{initial}}-HR_{\text{final}}, \end{array}$$

where *HR*_initial_ and *HR*_final_ are the resting heart rates before and after the optimized PE schedules. A greater Δ*HR* suggests a significant improvement in cardiovascular fitness.

#### 3.4.5 Weighted fitness function

To provide a comprehensive measure of overall health improvement, we developed a weighted fitness function. This function combines multiple health metrics, including BMI reduction, fitness improvement, calories burned, and heart rate reduction. The approach reflects the multidimensional nature of health outcomes associated with PE programs. By aggregating these metrics, the fitness function ensures the optimization process captures both immediate and long-term health benefits. It systematically addresses diverse health priorities.

PE time plays a critical role in influencing these metrics. Extended PE sessions offer more opportunities for structured physical activities. This leads to higher caloric expenditure and improved cardiovascular fitness. Conversely, shorter PE times may limit the effectiveness of these outcomes. Each optimization algorithm incorporates PE time as a decision variable. The algorithms adjust activity durations and frequencies to maximize health benefits within school scheduling constraints.

The rationale for assigning weights to these metrics is based on their relative importance in public health contexts. BMI reduction was given the highest weight (40%) because it addresses childhood obesity, a major risk factor for chronic diseases. Fitness improvement (30%) was prioritized for its contributions to cardiovascular health, muscular endurance, and overall fitness. Calories burned (20%) and heart rate reduction (10%) were included to reflect energy expenditure and cardiovascular improvements. These metrics were integrated into the following weighted fitness function:


(7) $$\begin{array}{l} f\left(x\right)=w_{1}\cdot \Delta \text{BMI}+w_{2}\cdot \Delta F+w_{3}\cdot \text{Calories Burned}+w_{4}\cdot \Delta HR, \end{array}$$

Here, *w*_1_, *w*_2_, *w*_3_, and *w*_4_ are weights that represent the relative importance of each metric. The weighting strategy aligns with the broader goal of maximizing health outcomes. It also provides flexibility to adapt to the needs of specific populations. By incorporating PE time as a decision variable, the algorithms dynamically optimize its allocation. This enhances the overall impact of PE schedules on public health goals.

#### 3.4.6 Optimal PE time

Optimal PE time is a key decision variable that represents the total hours allocated to physical education sessions each week (43–45). This metric ensures sufficient time is dedicated to structured physical activities while maintaining a balance with academic priorities. The optimization algorithms use PE time explicitly to calculate its impact on the weighted fitness function. This ensures that schedules align with global public health recommendations, such as the WHO's guideline for at least 60 minutes of moderate-to-vigorous physical activity (MVPA) daily for children and adolescents.

The weekly optimal PE time (*T*_optimal_) is calculated using Equation 8:


(8) $$\begin{array}{l} T_{\text{optimal}}=\frac{n\cdot t_{\text{session}}}{\text{week}}, \end{array}$$

where *n* is the number of PE sessions per week, *t*_session_ is the duration of each session in hours, and *week* denotes the total number of days (typically 5-7) in the schedule.

PE time directly influences other health outcomes. Longer PE times correlate with higher caloric expenditure and increased activity intensity. This supports energy balance and weight management. Sufficient PE time enables structured activities that improve muscular and cardiovascular endurance. Regular and prolonged sessions amplify the benefits of sustained physical activity, including reductions in resting heart rate.

Optimal PE time is central to the optimization algorithms. It allows activity durations and frequencies to be allocated effectively. This integration ensures schedules meet targeted health goals while remaining feasible for schools. By leveraging PE time, the algorithms balance health benefits with operational constraints. This enhances the overall effectiveness of PE programs in promoting public health.

### 3.5 Optimization algorithms

To optimize the PE schedules for enhancing public health outcomes, six widely recognized optimization algorithms were implemented: Genetic Algorithm (GA) (46), Particle Swarm Optimization (PSO) (47), Ant Colony Optimization (ACO) (48), Simulated Annealing (SA) (49), Differential Evolution (DE) (50), and Artificial Bee Colony (ABC) (51). Each algorithm was designed to maximize the health metrics, including BMI reduction, fitness improvement, caloric expenditure, and cardiovascular health. Table 5 shows the strengths of the selected optimization algorithms and their alignment with the optimization of PE schedules. Each algorithm targets specific aspects of PE program design and health metric optimization, thereby offering a comprehensive approach to enhancing public health outcomes. Additionally, Table 6 shows feature-wise comparison of optimization algorithms. It shows that ACO and DE are better optimization approaches over others.

**Table 5:** Benefits of selected optimization algorithms in PE program optimization.

**Algo**.	**Strengths**	**Alignment with PE and health metrics**
GA	Evolutionary approach for iterative refinement and global search capabilities.	Effective for BMI reduction by fine-tuning schedules for weight management.
PSO	Efficient optimization of interconnected variables through swarm intelligence.	Suitable for balancing activity intensity and session frequency to maximize caloric expenditure and fitness improvement.
ACO	Excels in solving allocation problems and finding optimal paths.	Ideal for determining time distribution across PE activities to improve fitness scores and energy balance.
SA	Escapes local optima by probabilistic acceptance of suboptimal solutions.	Useful for exploring diverse schedule options to enhance cardiovascular health (heart rate reduction).
DE	Handles multidimensional decision variables efficiently.	Effective for optimizing multiple health metrics (e.g., BMI, fitness score, and caloric expenditure) simultaneously.
ABC	Mimics natural foraging behavior for global optimization.	Useful for fine-tuning activity intensity levels to improve cardiovascular efficiency and energy expenditure.

**Table 6:** Feature-wise comparison of optimization algorithms.

**Algo**.	**Convergence speed**	**Local optima avoidance**	**Exploration vs. exploitation**	**Scalability**	**Computational complexity**	**Global search**	**Convergence stability**
GA	✓	✓	✓	✓	×	✓	✓
PSO	✓	×	✓	✓	✓	✓	✓
ACO	✓	✓	✓	×	✓	✓	✓
SA	×	✓	✓	×	✓	×	×
DE	✓	✓	✓	✓	✓	✓	✓
ABC	✓	×	✓	✓	×	✓	×

#### 3.5.1 Genetic Algorithm

The GA operates based on the principles of natural selection. It begins with an initial population of randomly generated solutions and iteratively evolves them over generations to improve their fitness. The main steps of GA are presented in Algorithm 1. By applying the GA to optimize school physical education (PE) schedules, it becomes possible to systematically allocate time and activities to maximize health benefits. The algorithm leverages the weighted customized fitness function in Equation 7, ensuring a focus on critical health metrics such as BMI reduction, caloric expenditure, and cardiovascular health. This targeted approach not only enhances individual health outcomes but also contributes to the long-term goal of improving public health through structured and evidence-based PE programs.

**Algorithm 1:**
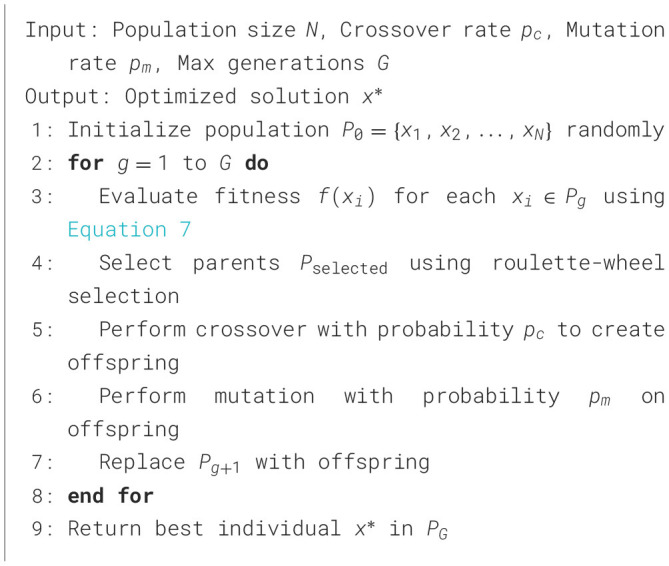
Genetic Algorithm.

#### 3.5.2 Particle Swarm Optimization

PSO mimics the social behavior of particles (solutions) in a swarm, collectively searching for the optimal solution. Algorithm 2 outlines the steps required to optimize the PE schedules using PSO. By employing the weighted customized fitness function in Equation 7, PSO evaluates and adjusts particle positions to maximize health outcomes such as fitness improvement, caloric expenditure, and heart rate reduction. The algorithm's iterative updates ensure convergence toward schedules that align with public health goals, promoting equitable and effective physical activity strategies in schools. This dynamic optimization process highlights PSO's capability to balance diverse health metrics while enhancing overall schedule efficiency.

**Algorithm 2:**
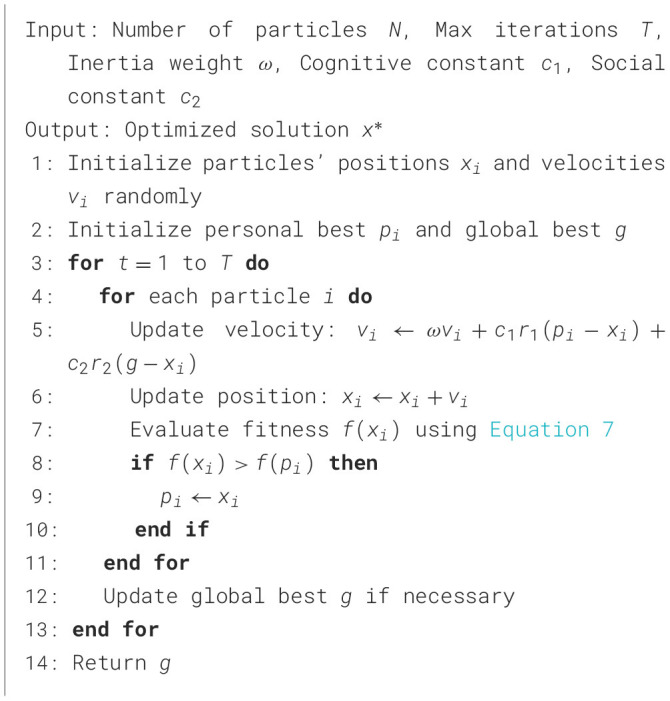
Particle Swarm Optimization.

#### 3.5.3 Ant Colony Optimization

Similarly, ACO is inspired by the foraging behavior of ants, where pheromone trails guide the search for optimal solutions. Ants probabilistically choose paths based on pheromone intensity (τ_*ij*_) and heuristic desirability (η_*ij*_), ensuring a balance between exploration and exploitation. Algorithm 3 presents the steps involved in optimizing PE schedules using ACO. By leveraging the weighted customized fitness function in Equation 7, ACO iteratively refines its solutions to improve metrics such as BMI reduction, fitness score improvement, and heart rate reduction. This approach demonstrates the algorithm's effectiveness in addressing complex scheduling challenges, aligning PE schedules with public health objectives.

**Algorithm 3:**
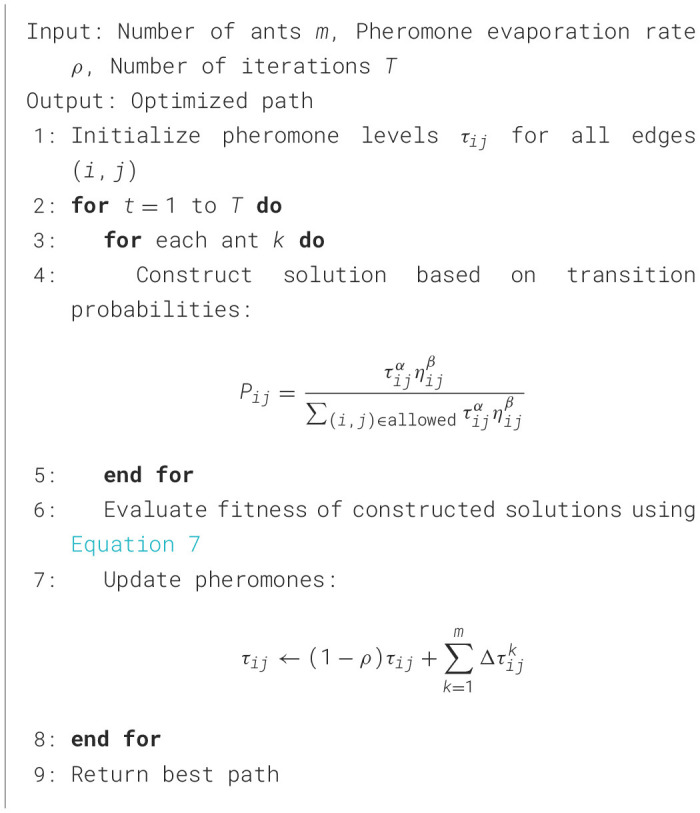
Ant Colony Optimization.

#### 3.5.4 Simulated Annealing

Simulated Annealing (SA) explores the solution space by allowing occasional acceptance of worse solutions to escape local optima, mimicking the annealing process in metallurgy. This probabilistic acceptance helps the algorithm avoid premature convergence and thoroughly search the solution space for optimal results. Algorithm 4 outlines the steps involved in applying SA to optimize PE schedules. By leveraging the weighted customized fitness function in Equation 7, SA evaluates solutions based on key health metrics such as BMI reduction and caloric expenditure. The temperature parameter gradually decreases. This ensures a focused search for optimal schedules that align with long-term public health goals. The probability of accepting a worse solution is given in Equation 9:


(9) $$\begin{array}{l} P=\exp\left(-\frac{\Delta E}{T}\right), \end{array}$$

where Δ*E* is the change in fitness, and *T* is the temperature. The temperature decreases over iterations in Equation 10:


(10) $$\begin{array}{l} T^{\left(t+1\right)}=\gamma \cdot T^{\left(t\right)}, \end{array}$$

where γ is the cooling rate.

**Algorithm 4:**
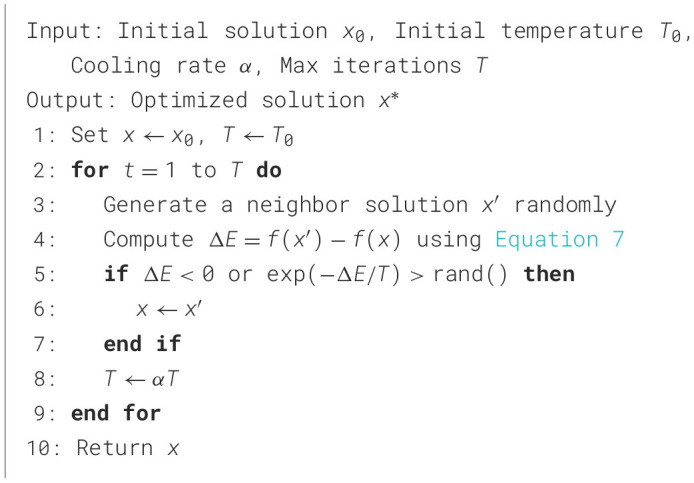
Simulated Annealing.

Algorithm 4 outlines the detailed steps involved in the SA process.

#### 3.5.5 Differential Evolution

Differential Evolution (DE) generates new solutions by combining existing ones using vector differences, ensuring a balance between exploration and exploitation of the solution space. This approach enables DE to effectively navigate complex optimization problems. In the context of optimizing PE schedules, DE applies its mutation and crossover mechanisms to iteratively refine solutions, guided by the weighted customized fitness function in Equation 7. Algorithm 5 details the steps required for DE to maximize health outcomes, such as BMI reduction, fitness score improvement, and caloric expenditure, ensuring schedules align with public health objectives. The algorithm's ability to adaptively explore solutions makes it a robust choice for enhancing PE program efficiency. A trial solution of DE can be created using Equations 11 and 12:


(11) $$\begin{array}{l} v_{i}=x_{r}1+F\cdot \left(x_{r}2-x_{r}3\right), \end{array}$$

(12) $$u_{i}=\{\begin{array}{ll} v_{ij} & \text{if }r<CR,\\ x_{ij} & \text{otherwise}, \end{array}$$

where *x*_*r*_1, *x*_*r*_2, *x*_*r*_3 are random solutions, *F* is the scaling factor, and *CR* is the crossover probability.

**Algorithm 5:**
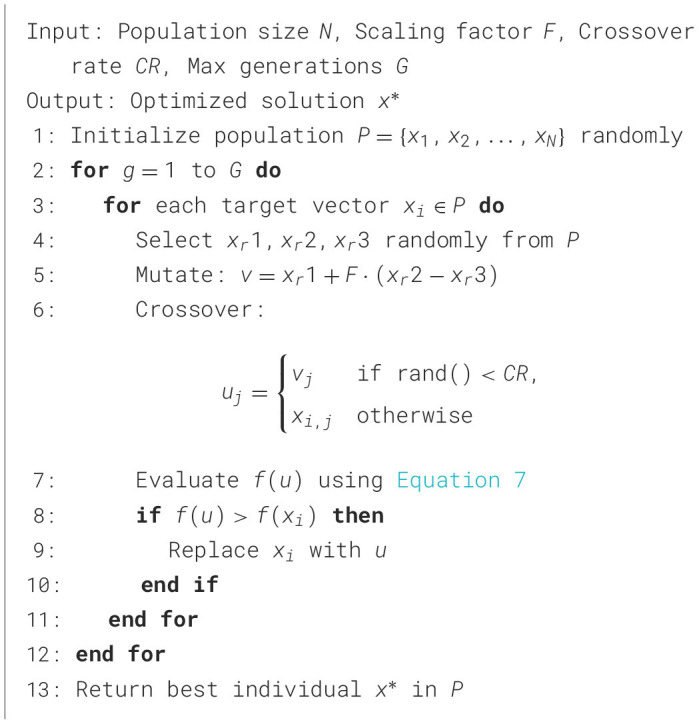
Differential Evolution.

#### 3.5.6 Artificial Bee Colony

Artificial Bee Colony (ABC) models the foraging behavior of honeybees, effectively mimicking their exploration and exploitation strategies to identify optimal solutions. In the context of optimizing PE schedules, ABC employs a collaborative approach among three types of bees: employed bees, onlooker bees, and scout bees. Each type plays a distinct role, contributing to the exploration of the solution space and refinement of potential solutions. The employed bees exploit known food sources (solutions), while onlooker bees evaluate the fitness of those solutions to make informed selections. Scout bees, on the other hand, introduce new solutions by abandoning poorly performing ones. Algorithm 6 illustrates the detailed step-by-step process of ABC, emphasizing its capability to utilize the weighted customized fitness function in Equation 7 for achieving enhanced public health outcomes through optimized PE schedules.

**Algorithm 6:**
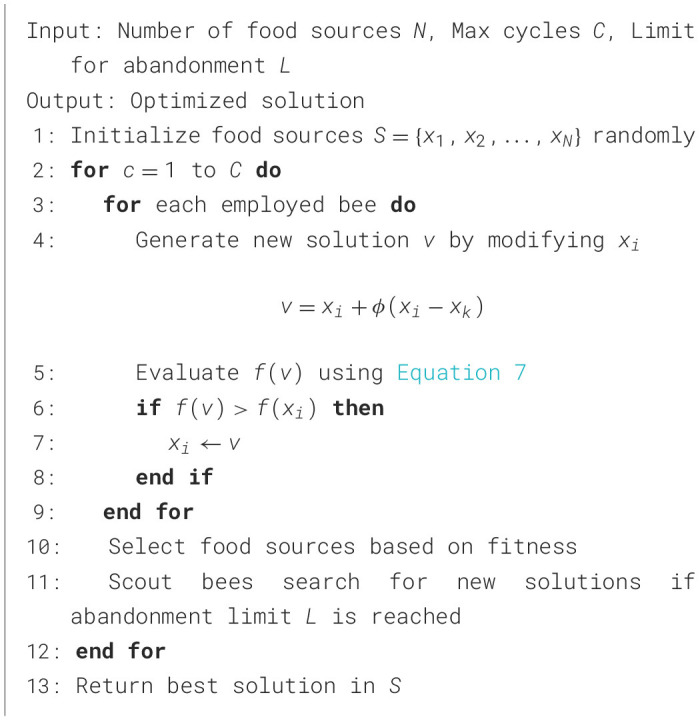
Artificial Bee Colony.

## 4 Results and discussion

This section presents a comprehensive evaluation of six popular optimization algorithms such as GA, PSO, ACO, SA, DE, and ABC in optimizing school PE schedules to improve long-term public health outcomes. These parameters were determined based on an extensive literature review to align with best practices in optimization studies and further refined using a trial-and-error approach. This iterative process ensured that the selected parameters achieved optimal balance between convergence speed, stability, and solution quality. The comparison focuses on five critical health metrics such as optimal PE time, reduction in BMI, improvement of fitness score, calories burned, and reduction of heart rate. Each algorithm was run for 200 iterations with a population size of 30 solutions. The fitness function was used to evaluate the effectiveness of the solutions, prioritizing health outcomes.

### 4.1 Experimental analysis

#### 4.1.1 Sensitivity analysis of fitness-function weights

Table 7 presents the results of the ACO-based sensitivity analysis that examines how small changes in fitness-function weights affect key health metrics. Increasing the weight assigned to BMI reduction by 5% enhances the BMI priority index by 15%. This prioritizes schedules with higher caloric expenditure and activity intensity. Conversely, reducing the weight by 5% results in a 15% decrease in BMI priority. This change indicates a shift away from BMI-focused interventions. These adjustments highlight the significant influence of weight modifications on the optimization outputs.

**Table 7:** ACO-based sensitivity analysis of fitness-function weight variations.

**Weight change (%)**	**BMI priority (index)**	**Caloric expenditure (kcal/session)**	**Fitness score (index)**	**Heart rate reduction (bpm)**
-5	85.0	315.0	105.0	8.24
0	100.0	350.0	100.0	8.00
+5	115.0	385.0	95.0	7.76

Changes in weight allocation also affect caloric expenditure outcomes. A 5% increase in BMI weight leads to a 10% rise in caloric expenditure, reaching 385 kcal/session. On the other hand, reducing the weight by 5% lowers caloric expenditure to 315 kcal/session, which is a 10% decline. This demonstrates a direct relationship between weight adjustments and energy expenditure outcomes in the schedules. The fitness score and heart rate reduction metrics reflect a different trend. Increasing BMI weights by 5% results in a 5% reduction in the fitness score and a slight decline of about 3% in heart rate reduction. These findings suggest that prioritizing BMI reduction may slightly reduce the emphasis on cardiovascular improvements.

#### 4.1.2 Convergence analysis

**Figure 2:**
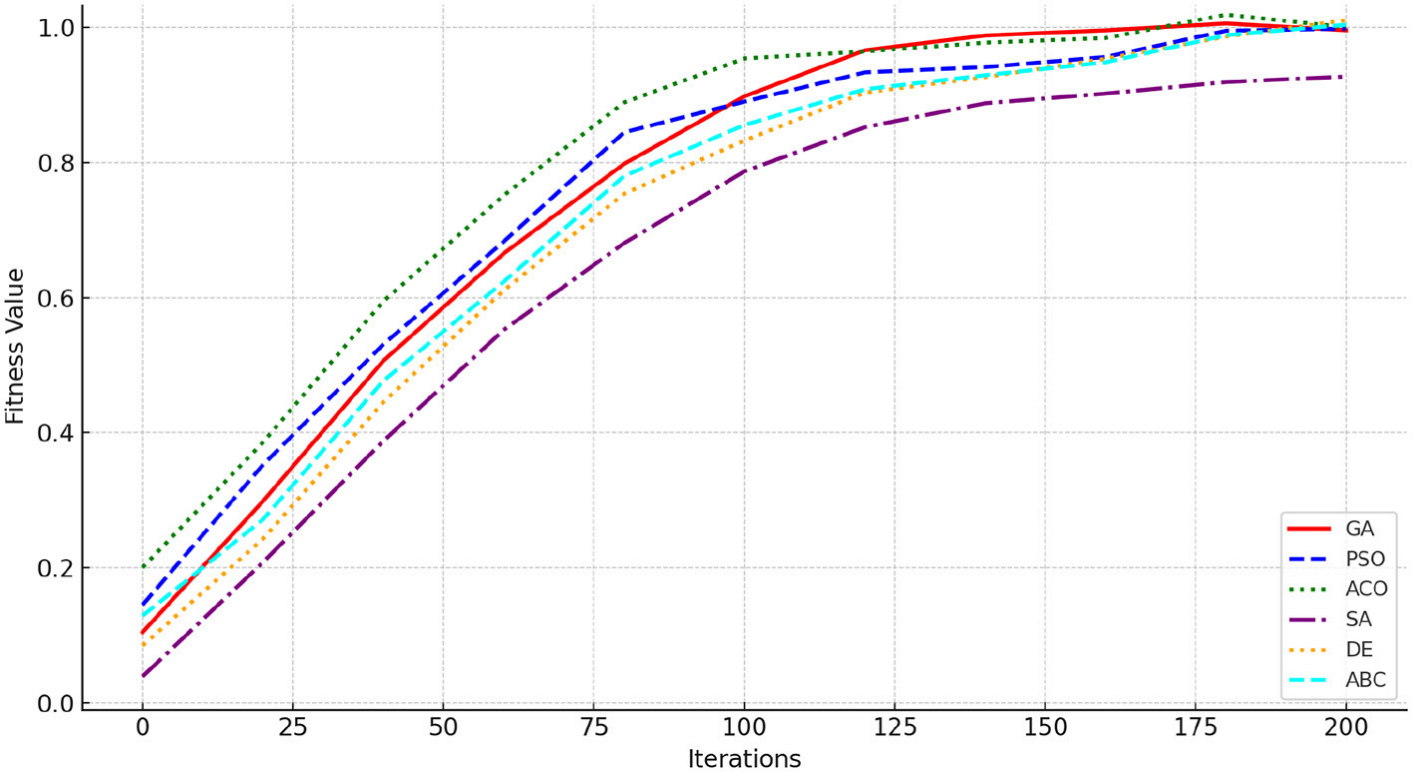
Convergence comparison of GA, PSO, ACO, SA, DE, and ABC algorithms.

Figure 2 illustrates the convergence behavior of six optimization algorithms such as GA, PSO, ACO, SA, DE, and ABC–toward the optimal fitness value. Each curve represents the fitness improvement over 200 iterations. ACO demonstrates the fastest and most consistent convergence, achieving the highest fitness value at around 9.91 hours of optimal PE time allocation and associated health metrics. DE and PSO follow closely, with steady improvement and robust performance across iterations. GA achieves competitive results but exhibits slower convergence compared to ACO. SA and ABC, while effective, converge more slowly and reach slightly lower fitness values. Therefore, ACO can be used an ideal choice for achieving optimal public health outcomes.

#### 4.1.3 Optimal PE time allocation

**Figure 3:**
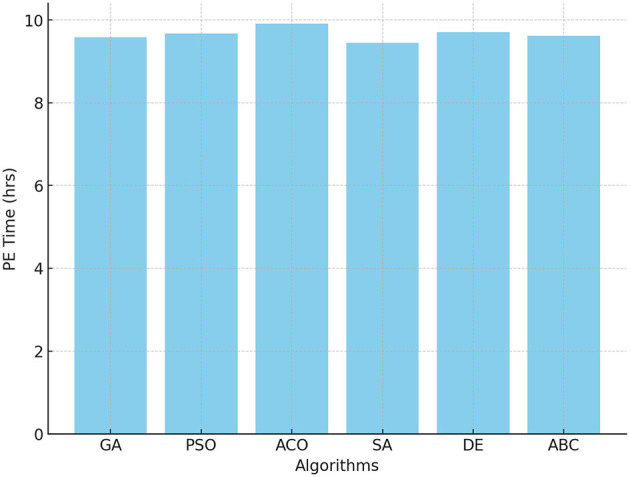
Optimal PE time across GA, PSO, ACO, SA, DE, and ABC algorithms.

PE time is a fundamental factor in promoting physical activity among students. Figure 3 indicates that ACO allocated the highest PE time of 9.91 hours / week, aligning with the strategy to maximize physical activity for health benefits. PSO and DE followed closely, with values of 9.67 and 9.71 hours, respectively. However, SA achieved the lowest PE time of 9.45 hours, likely due to its tendency to converge faster, but sometimes at suboptimal points.

#### 4.1.4 BMI reduction

**Figure 4:**
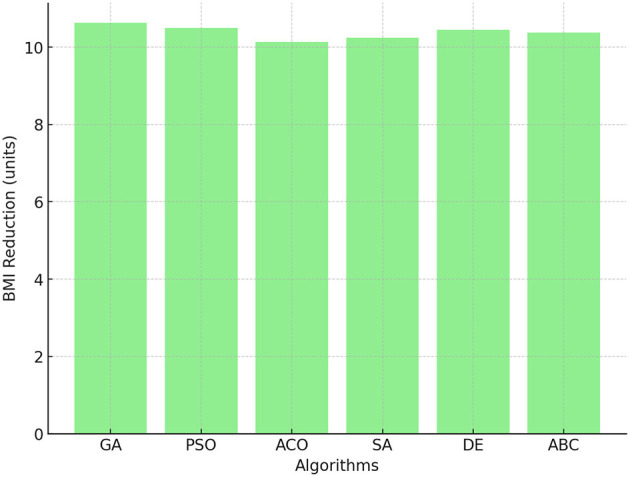
BMI reduction across GA, PSO, ACO, SA, DE, and ABC algorithms.

BMI reduction is a key indicator of effective weight management and physical activity. In Figure 4, it is found that GA achieved the most significant reduction in BMI, with an average of 10.63 units. This highlights its efficiency in optimizing schedules to combat obesity. The PSO and DE algorithms also performed well, achieving BMI reductions of < 10.50 units and < 10.45 units, respectively. Although ACO excelled in PE time, its performance in reducing BMI was slightly lower at 10.14 units. This result suggests that although longer PE time improves overall fitness, targeted strategies may be needed to achieve a greater reduction in BMI.

#### 4.1.5 Fitness score improvement

**Figure 5:**
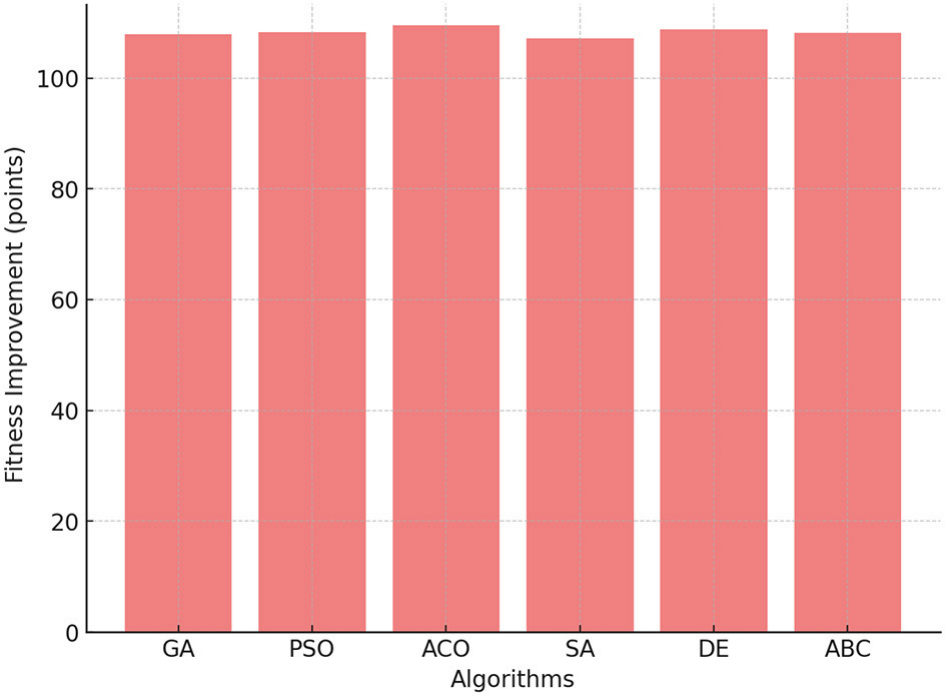
Fitness score improvement across GA, PSO, ACO, SA, DE, and ABC algorithms.

The improvement in fitness reflects the improvement in cardiovascular and muscular endurance due to physical activity. In Figure 5, ACO demonstrated the highest improvement in the fitness score, achieving an average of 109.5 points. This finding indicates that ACO effectively balances PE time and activity intensity, leading to significant improvements in fitness levels. PSO and DE followed closely, with improvements of 108.3 points and 108.8 points, respectively. GA performed well but fell slightly behind, achieving a fitness score of 107.9 points.

#### 4.1.6 Calories burned

**Figure 6:**
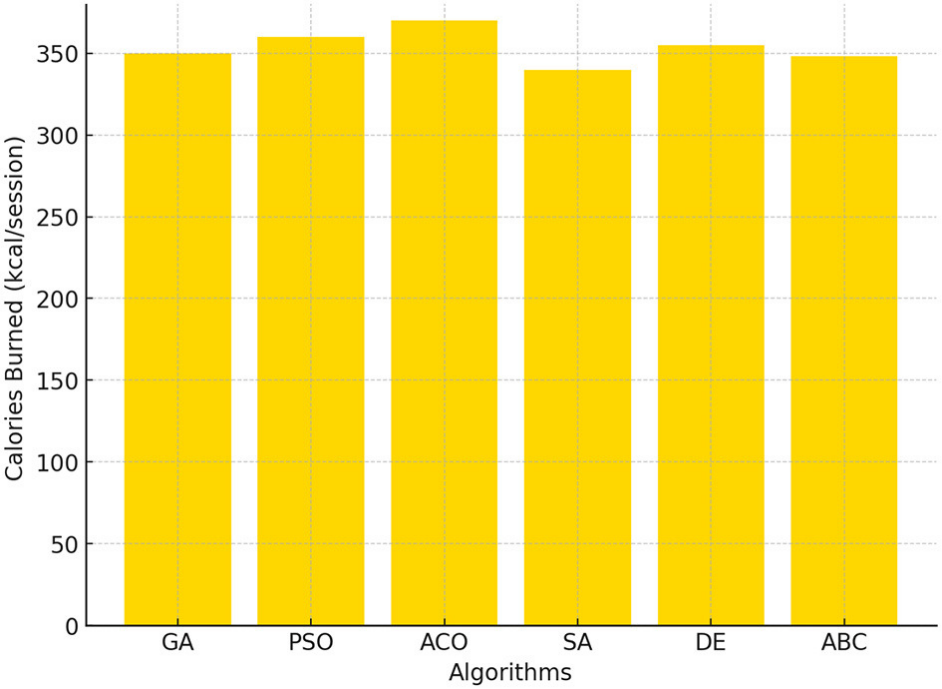
Calories burned across GA, PSO, ACO, SA, DE, and ABC algorithms.

The calories burned metric measures energy expenditure, which directly impacts weight management and overall health. In Figure 6, the ACO again emerged as the top performer, with 370 kcal burned per session. PSO and DE were closely followed with values of 360 kcal and 355 kcal, respectively. Meanwhile, SA achieved the lowest calorie burn of 340 kcal, consistent with its lower PE time allocation. The superior performance of ACO in this metric highlights its ability to maximize the intensity and duration of physical activity.

#### 4.1.7 Heart rate reduction

**Figure 7:**
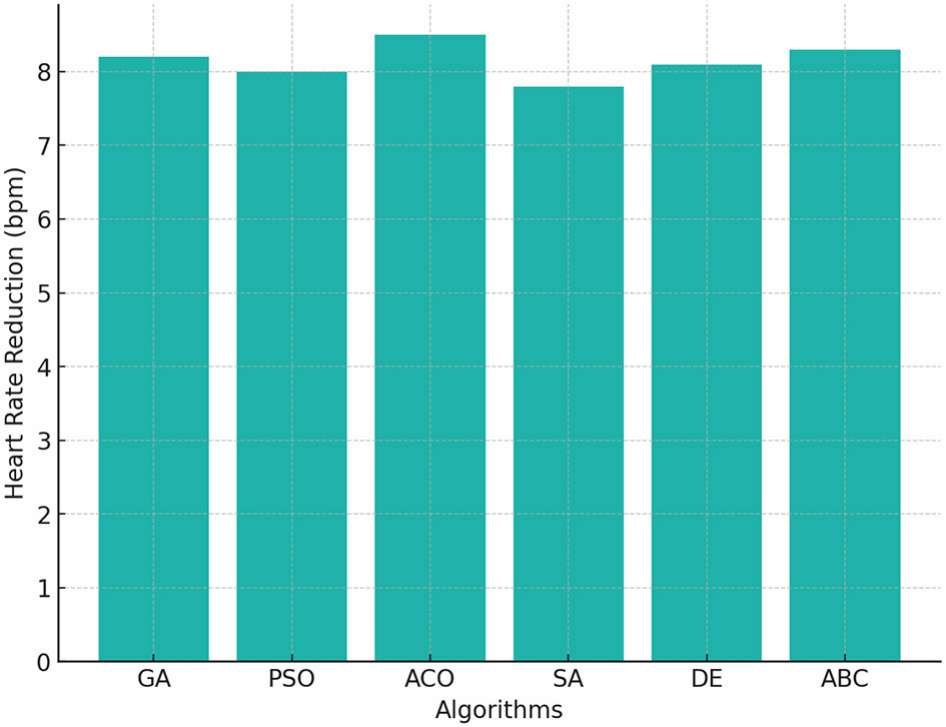
Heart rate reduction across GA, PSO, ACO, SA, DE, and ABC algorithms.

The reduction in heart rate is an indicator of improved cardiovascular fitness. Figure 7 shows that ACO achieved the highest heart rate reduction of 8.5 bpm, followed by ABC with 8.3 bpm and GA with 8.2 bpm. SA demonstrated the lowest reduction at 7.8 bpm, aligning with its overall lower performance in PE time and calorie burn. These results confirm that optimizing exercise program can significantly improve cardiovascular health, with ACO delivering the most balanced results.

#### 4.1.8 Summary of results

Table 8 summarizes the performance of GA, PSO, ACO, SA, DE, and ABC algorithms in five health metrics such as PE time (h), BMI reduction, fitness score, calories burned, and heart rate reduction. ACO emerged as the most balanced and effective algorithm, achieving the highest scores in PT time, fitness score, calories burned, and reduction of heart rate. GA excelled in reducing BMI, reflecting its efficiency in addressing weight-specific goals, particularly for obesity-focused interventions. PSO and DE offered robust and consistent performance in all metrics, positioning them as reliable alternatives for PE optimization.

**Table 8:** Summary of results across all metrics with confidence intervals.

**Algo**.	**PE time (hrs)**	**BMI reduction (units)**	**Fitness score (points)**	**Calories burned (kcal/session)**	**Heart rate reduction (bpm)**
GA	9.58 ± 0.12	10.63 ± 0.20	107.9 ± 1.5	350 ± 10	8.2 ± 0.2
PSO	9.67 ± 0.15	10.50 ± 0.18	108.3 ± 1.3	360 ± 12	8.0 ± 0.2
ACO	9.91 ± 0.10	10.14 ± 0.22	109.5 ± 1.0	370 ± 8	8.5 ± 0.1
SA	9.45 ± 0.18	10.25 ± 0.25	107.2 ± 1.8	340 ± 15	7.8 ± 0.3
DE	9.71 ± 0.14	10.45 ± 0.21	108.8 ± 1.2	355 ± 10	8.1 ± 0.2
ABC	9.62 ± 0.16	10.38 ± 0.19	108.1 ± 1.4	348 ± 11	8.3 ± 0.2

Meanwhile, SA recorded the lowest values in most metrics, indicating its limitations in maximizing activity duration and intensity. ABC showed moderate results, but performed better in heart rate reduction than SA and was comparable to PSO and DE in overall performance.

### 4.2 Public health implications of optimized PE schedules

The integration of optimization algorithms into school PE schedules presents transformative opportunities to improve public health outcomes. The impact of these interventions can be categorized as follows.

#### 4.2.1 Promotion of lifelong physical activity habits

Optimized PE schedules promote structured regular physical activity in children. This involvement helps cultivate lifelong habits. Active children are more likely to maintain an active lifestyle as adults. This reduces the risk of chronic diseases such as obesity, diabetes, and cardiovascular disorders. Implementing algorithms like ACO supports increased activity levels. Schools can thus meet the WHO's 60-minute daily exercise recommendation. These schedules also maximize participation in PE while balancing academics. This makes physical activity essential in the school curriculum.

#### 4.2.2 Reduction of chronic disease burden

Childhood is a critical period for preventing non-communicable diseases (NCDs). Optimized PE schedules contribute directly to weight management, improved cardiovascular health, and enhanced metabolic function. For instance, the results of this study demonstrate how ACO and GA effectively reduce BMI and improve heart rate. These are two key predictors of long-term health outcomes. By addressing these risk factors early, schools act as public health hubs. They reduce the burden on healthcare systems and mitigate the financial and social impacts of chronic diseases.

#### 4.2.3 Mental health and emotional wellbeing

Physical activity has well-documented benefits for mental health. These benefits include reductions in anxiety, stress, and depression. Optimized PE schedules ensure that students consistently access these benefits. These schedules include structured and engaging activities. Improved fitness levels and caloric expenditure are associated with higher self-esteem and emotional resilience in children. Algorithms such as ACO and PSO help achieve these fitness levels. Schools with optimized PE programs create supportive environments. These environments foster social interaction, teamwork, and a sense of belonging. All of these factors contribute to better mental health in students.

#### 4.2.4 Equitable health outcomes in underserved communities

Optimized PE schedules have significant public health benefits by addressing health disparities. In underserved communities, schools serve as critical points for promoting physical activity due to limited access to recreational facilities and organized sports. Customizing PE schedules to meet the needs of these communities ensures that children at risk of lifestyle-related diseases receive equitable health benefits. For example, extending PE times and using algorithms like ACO to incorporate high-impact activities can help resource-limited students achieve health outcomes similar to those in better-resourced settings.

#### 4.2.5 Cognitive and academic enhancements

Physical activity is closely related to improved cognitive function and academic performance. Optimized PE schedules maximize fitness and energy expenditure. This contributes to better memory, attention, and problem-solving abilities among students. Studies suggest that students who engage in regular physical activity perform better academically. This supports the argument that PE is not a distraction, but a catalyst for academic success. By integrating optimization algorithms into the PE scheduling, schools can balance physical and academic goals. This creates programs that improve learning outcomes and public health.

### 4.3 Implications for policymakers and stakeholders

The results of this study offer valuable information to policymakers, school administrators, and public health officials in designing evidence-based PE programs that maximize health benefits for students. Algorithms such as ACO and GA provide a robust framework for efficient allocation of time and resources, ensuring that students receive adequate physical activity despite competing academic demands.

#### 4.3.1 Evidence-based decision-making for schools

By leveraging optimization algorithms, schools can adopt data-driven approaches to improve PE scheduling. This ensures that resources are utilized effectively to achieve measurable health outcomes, such as reduced BMI, improved cardiovascular health, and improved fitness levels. Evidence-based interventions not only benefit students, but also empower schools to demonstrate accountability and efficacy in meeting public health goals.

#### 4.3.2 Alignment with public health goals

Optimized PE schedules align directly with broader public health objectives, including those established by the WHO and national health agencies. By prioritizing physical activity and improving fitness, schools help reduce the prevalence of non-communicable diseases (NCDs) and promote healthier lifestyles among future generations.

#### 4.3.3 Cost-effective health interventions

The adoption of algorithms such as ACO and GA enables schools to achieve significant health improvements without incurring substantial additional costs. Reduced obesity rates and improved fitness levels lead to a decrease in long-term healthcare expenses, alleviating the economic burden on families and healthcare systems. This cost-effectiveness is particularly valuable for resource-limited schools and communities.

#### 4.3.4 Addressing health disparities

Schools in underserved communities often face the challenge of providing equitable access to physical activity programs. Optimized PE schedules can be tailored to address these disparities, ensuring that all students, regardless of socioeconomic status, benefit from improved health outcomes. By promoting equitable access to quality PE programs, schools can contribute to closing the health gap and fostering social equity.

#### 4.3.5 Enhanced quality of life for students

Physical activity not only improves physical health, but also improves mental wellbeing and cognitive performance. Optimized PE schedules, guided by algorithms like ACO and GA, ensure that students develop essential life skills, including teamwork, discipline, and resilience. These benefits contribute to an improved quality of life, both during the school years and beyond.

### 4.4 Discussion

The comparative analysis highlights that ACO is the most effective algorithm for optimizing school PE schedules, achieving the highest results in PE time allocation, fitness improvement, calories burned, and heart rate reduction. Its ability to balance multiple health metrics makes it ideal for enhancing public health outcomes. In contrast, GA showed better performance for BMI reduction, making it particularly effective for weight-focused interventions aimed at reducing childhood obesity.

The differences in results across algorithms are highly relevant as they provide insights into the suitability of each algorithm for specific objectives. ACO's superior performance in optimizing PE time (9.91 hours/week) and associated metrics, such as calories burned (370 kcal/session) and heart rate reduction (8.5 bpm), underscores its role in holistic health improvements. This makes ACO an ideal choice for schools prioritizing cardiovascular health and overall energy expenditure. On the other hand, GA's exceptional performance in BMI reduction (10.63 units) highlights its effectiveness in tackling obesity, which is a critical public health challenge. These results suggest that GA is better suited for programs targeting weight-specific outcomes, such as interventions for overweight or obese students.

The consistent performance of PSO and DE across multiple metrics demonstrates their versatility. Both algorithms achieved competitive results in caloric expenditure and fitness improvement scores, indicating their potential for balanced public health improvements. Their reliability makes them suitable alternatives for schools seeking a comprehensive yet resource-efficient optimization approach. For instance, schools with limited resources can leverage PSO or DE to achieve satisfactory outcomes across multiple health metrics without compromising specific priorities.

In contrast, SA and ABC demonstrated slightly lower performance, particularly in metrics like PE time allocation and caloric expenditure. However, their exploratory nature and adaptability still make them valuable for scenarios requiring broader solution space exploration. For example, SA could be utilized in pilot studies or scenarios where constraints are less defined, allowing for flexible adjustments to PE schedules.

The differences among algorithms also reflect their underlying mechanisms and optimization strategies. ACO's ability to explore and exploit solutions simultaneously makes it highly effective for maximizing PE time allocation and activity intensity. GA's iterative refinement process aligns well with objectives requiring gradual improvements, such as weight reduction. PSO and DE excel in handling multidimensional variables, making them ideal for balancing various health metrics in complex schedules.

Optimized PE schedules directly contribute to improved public health outcomes by fostering an environment that prioritizes physical activity in schools. This is particularly significant given the rising rates of childhood obesity, cardiovascular disease, and metabolic disorders worldwide. The ability of algorithms such as ACO and GA to improve key health metrics provides schools with evidence-based tools to address these pressing health challenges.

Therefore, the differences in algorithm performance highlight the importance of selecting the right optimization strategy based on specific health goals. ACO and GA are ideal for schools with defined priorities in cardiovascular fitness and obesity reduction, respectively. Meanwhile, PSO and DE offer balanced solutions suitable for diverse health objectives. By understanding these differences, policymakers and educators can make informed decisions to maximize the impact of PE programs on student health and wellbeing.

### 4.5 Recommendations for health optimization

Table 9 presents a comprehensive overview of recommendations for health optimization derived from the performance of various optimization algorithms. Each recommendation area is linked to specific algorithms that excel in particular health metrics. It provides a distinction between the rationale behind each recommendation and practical examples of how they can be implemented in PE programs. Therefore, it highlights the practical utility of the applied analytic framework in designing evidence-based interventions for improving student health outcomes.

**Table 9:** Recommendations for health optimization based on algorithm performance.

**Recommendation**	**Algo**.	**Description**	**Example**
Enhanced PE time allocation	ACO	Allocate PE time similar to ACO's optimal value (9.91 hours/week), aligning with WHO's 60-minute MVPA guideline.	Incorporate diverse activities like aerobic exercises, strength training, and team sports to improve cardiovascular fitness and reduce sedentary behavior.
Targeted obesity interventions	GA	Leverage GA's performance for BMI reduction (10.63 units) to design calorie-intensive and strength-building PE activities.	Include HIIT and resistance-based exercises tailored for overweight and obese students.
Balanced activity schedules	PSO & DE	Use PSO and DE to balance activity types and durations, improving fitness scores and caloric expenditure.	Alternate between moderate aerobic activities (e.g., jogging) and vigorous team sports (e.g., basketball) for diverse physical benefits.
Cardiovascular health focus	ACO & ABC	Integrate sustained aerobic activities to achieve significant heart rate reduction (e.g., 8.5 bpm from ACO).	Include running or cycling in PE schedules and regularly monitor progress through heart rate assessments.
Resource-efficient scheduling	SA & ABC	Use SA and ABC to optimize schedules for schools with limited resources or facilities.	Design rotating activity groups to maximize facility utilization while maintaining program effectiveness.
Equitable health outcomes	All	Customize PE programs for underserved communities using high-impact activities with minimal equipment.	Include accessible exercises like bodyweight training or group aerobic routines to maximize engagement.

### 4.6 Potential limitations

While the proposed optimization models have demonstrated effectiveness in improving PE schedule design, there are several potential limitations that warrant further investigation.

1. **Scalability to larger datasets:** The current models were tested on datasets of moderate size (1,360 entries). Scaling these algorithms to handle significantly larger datasets may pose challenges related to computational efficiency and memory requirements. As datasets grow in size and complexity, the time required for convergence and the risk of overfitting may increase. Future research should explore optimization techniques that ensure scalability without compromising performance.
2. **Adaptability to demographic diversity:** The optimization models assume a generalized framework that may not fully account for the nuanced needs of diverse demographic groups. Factors such as age, gender, and socioeconomic background can influence the effectiveness of PE programs. Developing demographic-specific parameter tuning or incorporating adaptive learning algorithms could enhance the models' applicability across varied populations.
3. **Geographic and cultural contexts:** The models have yet to be validated in geographically or culturally diverse settings. PE program effectiveness can vary based on local infrastructure, climate, and cultural attitudes toward physical activity. Future studies should test the adaptability of the optimization algorithms in different geographic and cultural contexts to ensure universal applicability.
4. **Computational complexity:** Some of the proposed algorithms, such as ACO and GA, involve intensive computational processes, especially when optimizing multiple health metrics simultaneously. This complexity may limit their feasibility for real-time or resource-constrained applications. Simplified or hybrid models that balance computational demands and optimization performance could address this limitation.
5. **Resource constraints:** Teacher availability, facility capacity, and budget limitations are critical factors that could affect the practical implementation of optimized PE schedules. These constraints may limit the scalability and adaptability of the proposed model. Future work should incorporate these variables into the optimization process, allowing the model to adapt schedules to the available resources while maintaining health outcomes.

## 5 Conclusion and future directions

### 5.1 Conclusion

Optimizing school physical education (PE) schedules is crucial for enhancing public health outcomes, particularly among school-aged children. Therefore, in this study, a weighted fitness function is developed to evaluate health fitness scores. This function integrates multiple health metrics such as BMI reduction, fitness improvement, calories burned, and heart rate reduction. Six optimization algorithms such as Genetic Algorithm (GA), Particle Swarm Optimization (PSO), Ant Colony Optimization (ACO), Simulated Annealing (SA), Differential Evolution (DE), and Artificial Bee Colony (ABC) optimization algorithms are utilized to optimize PE schedules based on the designed weighted fitness function. Using a dataset of 1,360 student entries, the study incorporates health metrics such as BMI reduction, fitness score improvement, caloric expenditure, and heart rate reduction into a weighted fitness function for optimization. The results show that ACO achieved the highest allocation of PE time (9.91 hours / week), the most significant caloric expenditure (370 kcal / session) and the greatest reduction in heart rate (8.5 bpm). GA excelled in the reduction of BMI, achieving a decrease of 10.63 units. These analysis reveals that the transformative potential of optimized PE schedules in reducing the burden of lifestyle-related diseases, promoting equitable health outcomes, and supporting cognitive and mental wellbeing. Finally, recommendations are provided for policy makers and stakeholders to implement data-driven PE programs that maximize long-term public health benefits.

### 5.2 Future directions

This section outlines potential areas for further improvement and practical implementation challenges. These directions aim to build on the current work, enhancing its scalability, applicability, and real-world impact.

1. **Scalability to larger datasets:** Future work can explore the scalability of the proposed optimization algorithms to handle larger datasets. This includes evaluating computational efficiency and performance when applied to datasets with diverse demographic, geographic, and health characteristics.
2. **Hybrid optimization models and machine learning models:** Hybrid optimization models, combining multiple algorithms or integrating advanced machine learning techniques, could further improve the efficiency and accuracy of PE schedule optimization. For instance, combining the exploration capabilities of ACO with the local refinement strength of GA may yield better results.
3. **Demographic-specific challenges:** Addressing the unique needs of different demographic groups, including age, gender, socioeconomic status, and geographic location, remains a critical area. Customized optimization strategies can be developed to ensure equitable access and effectiveness of PE programs across diverse populations.
4. **Real-world implementation and validation:** Practical implementation of the proposed methods in real-world school settings would provide valuable insights into their feasibility and effectiveness. Collaborating with educational institutions to pilot these optimized schedules and monitor their long-term health impacts could bridge the gap between research and practice.

## Data Availability

The raw data supporting the conclusions of this article will be made available by the authors, without undue reservation.
